# The Role of Inflammasomes in Osteoarthritis and Secondary Joint Degeneration Diseases

**DOI:** 10.3390/life12050731

**Published:** 2022-05-13

**Authors:** Samo Roškar, Iva Hafner-Bratkovič

**Affiliations:** 1 Valdoltra Orthopaedic Hospital, Jadranska Cesta 31, SI-6280 Ankaran, Slovenia; samo.roskar@ob-valdoltra.si; 2 Faculty of Medicine, University of Ljubljana, Zaloška 9, SI-1000 Ljubljana, Slovenia; 3 Department of Synthetic Biology and Immunology, National Institute of Chemistry, Hajdrihova 19, SI-1000 Ljubljana, Slovenia; 4 EN-FIST Centre of Excellence, Trg OF 13, SI-1000 Ljubljana, Slovenia

**Keywords:** inflammation, inflammasome, osteoarthritis, rheumatoid arthritis, gout, therapy, inflammatory joint disease, NLRP3, caspase-1, CARD8

## Abstract

Osteoarthritis is age-related and the most common form of arthritis. The main characteristics of the disease are progressive loss of cartilage and secondary synovial inflammation, which finally result in pain, joint stiffness, and functional disability. Similarly, joint degeneration is characteristic of systemic inflammatory diseases such as rheumatoid arthritis and gout, with the associated secondary type of osteoarthritis. Studies suggest that inflammation importantly contributes to the progression of the disease. Particularly, cytokines TNFα and IL-1β drive catabolic signaling in affected joints. IL-1β is a product of inflammasome activation. Inflammasomes are inflammatory multiprotein complexes that propagate inflammation in various autoimmune and autoinflammatory conditions through cell death and the release of inflammatory cytokines and damage-associated molecule patterns. In this article, we review genetic, marker, and animal studies that establish inflammasomes as important drivers of secondary arthritis and discuss the current evidence for inflammasome involvement in primary osteoarthritis. The NLRP3 inflammasome has a significant role in the development of secondary osteoarthritis, and several studies have provided evidence of its role in the development of primary osteoarthritis, while other inflammasomes cannot be excluded. Inflammasome-targeted therapeutic options might thus provide a promising strategy to tackle these debilitating diseases.

## 1. Introduction

Osteoarthritis (OA) is the most common chronic degenerative joint disease, with an estimated age-standardized prevalence of up to 4%, without any apparent underlying reason [1,2,3]. Patients with OA suffer from joint pain, stiffness, and functional disabilities that considerably influence their life quality [3,4]. Major risk factors for the disease are considered to be increasing age and obesity. The disease is more prevalent in women. Among other important risk factors are infectious joint disease, injury, joint mechanical stress, and certain metabolic disorders [2,4,5]. Primarily affected joints are knees, hips, spine, and hand joints [2]. Characteristic radiological features of the disease are joint space narrowing, subchondral bone sclerosis, subchondral cysts, and osteophyte formation [4]. The pathogenesis of the disease is associated with progressive loss of articular cartilage and changes in subchondral bone and joint-related structures such as menisci, synovial membrane, ligaments, and periarticular muscle [6]. Cartilage degeneration leads secondarily to changes in subchondral bone and synovial inflammation [2]. The cartilage is a highly specialized connective tissue that is an avascular structure composed of chondrocytes and the extracellular matrix [7]. Among the matrix part, the major constituent substances are water, collagens, and proteoglycans [7]. The cartilage’s nutritional supply is primarily dependent on the synovial membrane. It is well established that inflammation in the synovial membrane plays a key role in the pathogenesis of OA [6]. The damaged chondrocytes release their content, including damage-associated molecular patterns (DAMPs), in joint space, which results in further propagation of inflammatory processes in the synovial membrane [8]. The inflamed synovial membrane further propagates the chondrocyte damage with secreted factors. In contrast to primary OA, with a poor understanding of the underlying causes, the underlying mechanisms are considerably more determined in the case of rheumatoid arthritis (RA), gouty arthritis, and calcium phosphate and pyrophosphate arthritis (reviewed in [9]). RA affects around 1% of the population worldwide; it is driven by chronic synovial inflammation, progressing to irreversible cartilage degradation and, finally, joint destruction [10]. Gout is reported to be present in up to 7% of the population worldwide; it is a disease caused by the deposition of monosodium urate crystals in articular and non-articular structures [11,12]. In all types of secondary OA, it was shown that the inflammatory processes include inflammasome activation. Within this review, we are going to discuss the current state-of-the-art in inflammasome involvement in secondary arthritis and how this translates to the potential of inflammasome involvement in primary osteoarthritis. We will end the review with a description of inflammasome-targeted approaches and their potential for treatment of arthritis.

## 2. The Inflammasomes, Multifacial Protein Inflammatory Complexes

**Figure 1:**
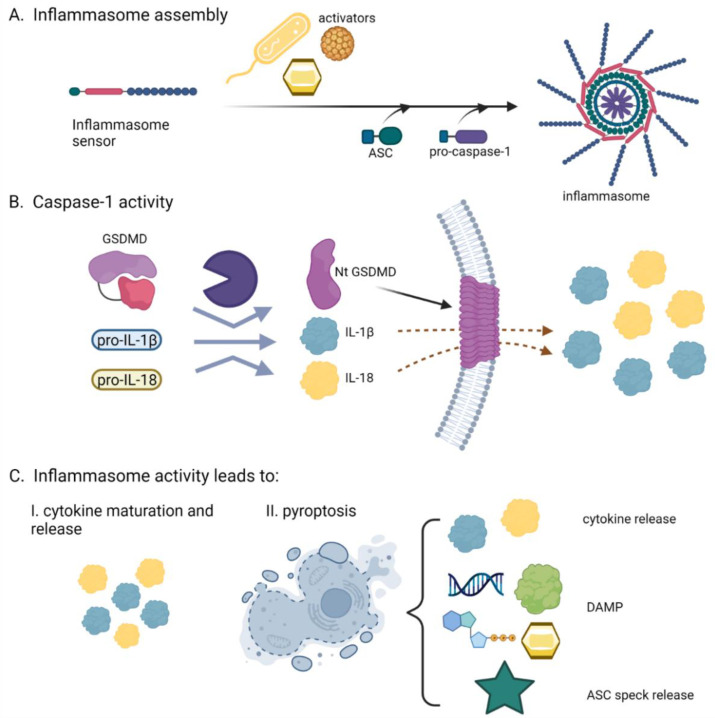
Scheme of inflammasome activation. (**A**) Inflammasome formation: upon activation, the inflammasome sensor forms a complex that recruits adaptor ASC and pro-caspase-1. Caspase-1 is activated within the inflammasome. (**B**) Active caspase-1 cleaves its substrates gasdermin D, pro-IL-1β and pro-IL-18. N-terminal gasdermin D forms pores in the plasma membrane that enable the release of smaller proteins, such as the mature form of IL-1β. (**C**) Important effects of inflammasome activation are cytokine release and pyroptosis. The latter perpetuates inflammation via additional cytokine release, DAMP release, and also the release of ASC that are active in the extracellular environment.

Since the discovery of inflammasomes two decades ago [13], these cytosolic protein inflammatory devices have been connected to various diseases. In this chapter, we first introduce inflammasomes and later describe how particular inflammasomes are activated. Inflammasomes are assembled upon the activation and likely oligomerization of the sensor protein, which recruits further molecules. While inflammasome sensors containing a caspase activation and recruitment domain (CARD) are able to recruit pro-caspase-1 molecules directly via sensor^CARD^-pro-caspase-1^CARD^ domain interactions, inflammasome sensors that contain a pyrin domain (PYD) will first recruit bidomain adaptor ASC composed of PYD and CARD domains. Adaptor ASC polymerizes via PYD domains, and pro-caspase-1 molecules bind to ASC filaments via ASC^CARD^-pro-caspase-1^CARD^ interactions [14] (Figure 1A). Inflammasome-mediated self-activation of caspase-1 leads to the proteolytic activation of proinflammatory cytokines interleukin (IL)-1β and IL-18. Cytokines IL-1β and IL-18 are members of the IL-1 cytokine family. By binding to their specific receptors, they induce intracellular signaling pathways: NF-κB, JNK, and p38 MAPK kinase signaling that lead to the production of IL-6, IL-8, and also pro-IL-1β [15,16] and IFN-γ [17]. Additionally, IL-1 and IL-18 signaling pathways lead to the production of catabolic enzymes such as matrix metalloproteinases. Caspase-1 cleaves gasdermin D, and this cleavage enables the N-terminal domain to form pores in the plasma membrane [18,19,20,21,22,23]. Gasdermin D pores, with an inner diameter of around 22 nm [24], enable the release of smaller proteins [25,26] and, with the help of Ninjurin-1, lead to lytic cell death and pyroptosis [27]. Upon pyroptosis, multiple DAMPs are released. An example of inflammasome-specific DAMP is ASC speck that can perpetuate inflammation by caspase-1 activation in the extracellular space [28,29,30] (Figure 1).

Several known inflammasome sensors, NLRP1, NLRP3, and NAIPs/NLRC4, are members of a nucleotide-binding and leucine-rich repeat receptor family. In spite of belonging to the same family, their activators and activation mechanisms are very different. The NLRP3 inflammasome is one of the most studied inflammasomes, particularly due to its likely involvement in versatile conditions, including autoinflammatory and rheumatic diseases. It also requires two signals for its activation. During the priming stage, NLRP3 and pro-IL-1β are expressed and NLRP3 is, by posttranscriptional modifications, prepared to respond to the second signal [31,32,33,34]. The priming stage is rather ill-defined and might depend on cell type and species, but it is nevertheless necessary for inflammasome activation (reviewed in [35]). Diverse compounds and processes contribute to the second signal, including damage-associated molecular patterns (DAMPs): adenosine triphosphate, aggregates, and crystals of different molecular species. The versatility of NLRP3 triggers suggests their indirect action, and most NLRP3 triggers have been shown to induce an efflux of intracellular K^+^ [36,37]. Many studies have suggested that NLRP3 senses perturbation in cell homeostasis, particularly when organelles are damaged (reviewed in [38]). Of particular interest is the destabilization of lysosomes and reactive oxygen species that is caused by aggregates such as monosodium urate and silica crystals [39,40,41], which will be described in detail later. In the inactive state, NLRP3 was recently discovered to be present as an inactive oligomer [42,43]. Upon activation, NLRP3 binds NEK7 [44,45,46], and this interaction forces NLRP3 to a semi-activated state [47]. How exactly NLRP3 is inflammasome activated is currently still unclear.

The NAIP/NLRC4 inflammasome is activated by the bacterial ligand flagellin and the rod and needle proteins of the type III secretion system when encountered in the cytosol. Those bacterial pathogen-associated molecular patterns (PAMPs) bind to the members of the NAIP family [48,49,50,51]. Ligand-activated NAIPs bind dormant NLRC4 molecules and induce oligomer formation [52,53] that serves either direct pro-caspase-1 activation through CARD–CARD interaction or indirect activation through ASC speck formation.

In contrast to the direct activation of the NAIP/NLRC4 inflammasome, NLRP1 is activated by functional degradation according to the N-end rule quality control pathway. NLRP1, in a way, senses pathogen activity, such as protease activity of bacterial and viral proteases [54,55,56,57] and ubiquitin ligases [58] that lead to NLRP1 N-terminus degradation. Human NLRP1 and mouse NLRP1b contain a function-to-find-domain (FIIND) followed by a CARD domain at the C-terminus. After degradation of the N-terminal part, the C-terminal CARD domain forms an inflammasome by recruiting ASC and pro-caspase-1. It has also been shown that dipeptidyl peptidases DPP8/9 inhibit NLRP1 inflammasome activation by disabling CARD domains to form the seed for inflammasome formation. Inhibition of DPP8/9 by Val-boro-pro (VbP) thus activates human and mouse NLRP1 [59]. A similar mechanism of activation is employed by CARD8, a protein that relates to NLRP1′s C-terminus as it is composed of FIIND and CARD domains. DPP8/9 inhibitors are able to induce CARD8 inflammasome formation [60] as well as proteolytic destabilization by N-terminal cleavage by HIV protease [61]. CARD8 does not engage ASC for pro-caspase-1 recruitment [62]. Interestingly, CARD8 has a dual role in inflammasome activation. Besides being an inflammasome-forming sensor by itself, it has also been shown to negatively regulate other inflammasomes, particularly NLRP3 [63].

AIM2 is not a member of the NLR receptor family but has been shown to be crucial for the recognition of cytosolic DNA and subsequent inflammasome formation [64,65,66,67]. AIM2 can, via its HIN domain, bind versatile DNAs of bacterial, viral, mitochondrial, host, and artificial DNA when encountered within the cytosol. Binding to DNA will induce PYD domains of several AIM2 molecules to recruit adaptor ASC and facilitate the self-processing of pro-caspase-1 molecules.

Pyrin belongs to the tripartite motif-containing (TRIM) family with the N-terminal PYD domain. Pyrin is not activated by direct binding of activators but senses the inactivation and decreased activity of host defense enzymes such as RhoA (a small Rho guanosine triphosphatase) [68]. The mechanism of activation is indirect. Decreased activity of Rho GTPases leads to decreased activity of PKN1/2 kinases that phosphorylate pyrin, keeping its inactive conformation by the binding of 14-3-3- proteins. The loss of protective posttranslational modifications leads to pyrin inflammasome activation [69,70].

In addition to the above-described canonical activation, mutations in genes encoding NLRP3, NLRP1, NLRC4, and pyrin induce constitutive inflammasome formation that underlies genetic autoinflammatory diseases. Constitutive NLRP3 activation causes cryopyrin-associated periodic syndromes: familial cold autoinflammatory syndrome, Muckle–Wells syndrome, and neonatal-onset multisystem inflammatory disease [71]. Constitutively active pyrin inflammasomes cause pyrin-associated autoinflammation with neutrophilic dermatosis (PAAND) [72] and familial Mediterranean fever (FMF) [70]. Mutations in the gene encoding NLRC4 cause versatile inflammatory syndromes, including autoinflammation with infantile enterocolitis [73]. Mutations in the gene for NLRP1 underlie multiple self-healing palmoplantar carcinoma and familial keratosis lichenoideschronica [74].

Inflammasomes can be assembled upon encountering various triggers of pathogen or host origin. We described the mechanisms of their activation as they are currently understood. In the following chapters, we plan to review studies that provide evidence for inflammasome involvement in primary and secondary osteoarthritis.

## 3. Inflammasome Involvement in Secondary Osteoarthritis

There are several levels of evidence for inflammasome involvement in secondary arthritis, such as rheumatoid arthritis, gout, and pseudogout. In vitro studies and animal models have provided mechanistic advances in defining DAMPs such as monosodium urate (MSU) crystals as the activators of NLRP3 inflammasome. In gout, the accumulation of monosodium urate crystals happens inside the joint. MSU crystals were shown to be a strong trigger for NLRP3 inflammasome activation, the IL-1β release, and subsequent chondral damage [40]. Similar findings were shown for calcium phosphate or pyrophosphate crystals that accumulate in pseudogout [40]. Crystals and aggregates, such as amyloids, activate the NLRP3 inflammasome through reactive oxygen species (ROS) formation, lysosome destabilization, and K^+^ efflux [36,39,40,75,76]. Crystals first need to be phagocytosed. They end up in lysosomes, where the failure of their degradation leads to lysosome destabilization [39,75,76], which, in turn, activates NLRP3. The majority of NLRP3 inflammasome triggers, including MSU crystals, were shown to induce trans Golgi network disassembly [77]. While cytokine IL-1β and IL-18 secretion in response to crystals depends on the NLRP3 inflammasome, necrotic cell death does not depend on inflammasomes and gasdermin D [76,78]. In RA, bone destruction leads to increased local concentrations of Ca^2+^ and phosphate. Fetuin-A binds Ca^2+^ to form calciprotein particles (CPP) that are colloidal in nature. Excessive Ca^2+^ induces calcium-sensing receptor-mediated macropinocytosis of CPP that leads to NLRP3 inflammasome-mediated IL-1β maturation that does not induce lysosome destabilization in contrast to MSU crystals [79]. Mechanistic and animal studies provide strong support for inflammasome involvement in secondary arthritis. The involvement of inflammasome components and their effector functions in mouse models of RA, such as the antigen-induced arthritis model, has been proven using various knockout models such as ASC-deficient mice [80], and a recent study suggested that ASC specks that are released from pyroptotic cells importantly contribute to joint inflammation [30]. Additionally, enhanced expression of inflammasome components in A20-knockout mice predisposes those mice to a RA-prone phenotype that is improved upon the deletion of NLRP3, caspase-1 or IL-1 receptor-encoding genes [81].

Another level of evidence comes from human genetic data that suggest a strong association of inflammasome activity in different types of secondary arthritis. As recently reviewed by Spel [9], several SNPs in the genes encoding NLRP3, NLRP1, IL-1β, and IL-18, which lead to increased expression levels, were connected to the development of rheumatoid arthritis, gout, ankylosing spondylitis, and juvenile idiopathic arthritis. Polymorphisms in *CARD8* leading to decreased expression of CARD8 were also connected to the severity of rheumatoid arthritis and gout (reviewed in [9]); however, in this case, CARD8 does not act as an inflammasome sensor but rather as a potential inhibitory regulator of NLRP3 [82]. In RA, for example, gene expression of the inflammasome-related constituents (NLRP3, ASC, caspase-1, IL-1β, and IL-1R) was significantly higher in peripheral blood macrophages of patients with RA compared to healthy controls [83,84,85]. Furthermore, it has been shown that single polymorphisms at the NLRP3 are associated with RA susceptibility and correlate with higher disease activity. NLRP3 polymorphism combined with CARD8 polymorphism is associated not only with greater susceptibility but also greater severity of the disease [86]. It is also known that several polymorphisms in inflammasome-related genes (NLRP3, IL-1β, CARD8) are associated with a greater risk of developing gout [87,88,89].

The third level of evidence comes from the following inflammation in joints. In RA patients’ sera, a prominent increase in TNF, IL-6, and IL-1β concentrations compared to healthy controls was identified [9]. Particularly the IL-1β levels were increased in both serum and synovial fluid samples of patients with RA [90]. Higher levels of IL-1β were associated with higher IL-18 levels in synovial fluid samples, which supports the hypothesis of the inflammasomes’ prominent role in the pathogenesis of RA [90], and NLRP3 inflammasome activation was correlated to Th17 differentiation in RA patients [91]. Recently, it has been shown that the anti-citrullinated protein antibodies (ACPA), which are informative RA biomarkers, activate AKT/NF-κB signaling pathway that results in inflammasome priming [92]. Similar to RA patients, the serum and synovial fluid samples of the patients with gout were detected to contain higher levels of NLRP3, caspase-1, IL-1β, and IL-18 [93,94].

From the cellular point of view, it has been shown that the chronic synovitis characteristic of RA is closely related to the disruption of the synovial lining layer integrity. The inflammation-based destruction of tight junctions between the lining macrophages is considered to allow the infiltration of the intraarticular space primarily with fibroblasts that exhibit strong proinflammatory gene expression. Furthermore, the secreted proinflammatory cytokines recruit additional inflammatory cells from the bloodstream that propagate the initiated process of joint destruction [95,96]. It has already been shown that the synovial fibroblasts, as the most abundant cells in the synovial membrane, release proinflammatory cytokines such as IL-1β and TNFα [97,98].

Mechanistic, genetic, and biomarker studies establish a strong link between pathological inflammasome activation, leading to IL-1β and IL-18 secretion and the progression of RA and gout.

## 4. Inflammasome Involvement in Primary Osteoarthritis

There is significant evidence that inflammasomes play an important role in inflammatory joint diseases such as RA and gout, as presented in the previous section. On the contrary, a limited amount of literature is available to explain the direct role of inflammasomes in the development of OA. However, recent studies suggest that inflammasomes are involved. Several types of inflammasome triggers are present in OA joints. Calcium phosphate crystals were found in the knee and hip joints of patients with primary OA [99]. It has been shown that the level of uric acid in synovial fluid correlates to the synovial fluid level of IL-1β and IL-18 and the severity of OA [100]. The MSU crystals, calcium pyrophosphate, and calcium phosphate are well-established inflammasome activators [101]. Hydroxyapatite crystals can be found in OA joints [76]. Their mechanism of NLRP3 inflammasome activation involves K^+^ efflux, ROS, and lysosome destabilization, as described above for other particulate triggers [76].

**Figure 2:**
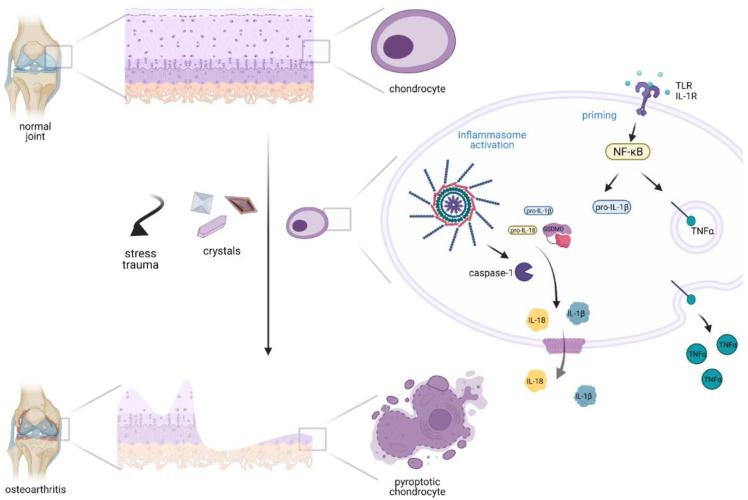
Scheme of proposed inflammasome involvement in primary osteoarthritis. Different external stimuli such as mechanical stress, ROS, hyperlipidemia, trauma, MSU, calcium pyrophosphate, and calcium phosphate crystals activate TLR receptors and mediate the inflammasome assembly. Activation of the inflammasome leads to activation of caspase-1, which cleaves GSDMD, pro-IL-1β, and pro-IL-18. The N-terminal GSDMD forms a transmembrane pore, which is one possible transport route of active IL-1β and IL-18 into extracellular space. Furthermore, TLRs activate the nuclear transcription factor NF-κB, which promotes the transcription of transmembrane pro-TNFα that is released from the cell as TNFα. IL-1β, IL-18, and TNFα provide further activation signals and thus promote further chondrocyte death and propagation of the process further to the cells in the synovial membrane.

Inflammasome priming leads to the production of inflammatory cytokines such as TNF-α and pro-IL-1β. The expression of the sensor NLRP3 is increased during the priming step [32,35]. The level of NLRP3 was shown to be more than five-fold higher in synovial membrane cells in OA than in normal controls [76,102]. The synovial fibroblasts, which are the most abundant cells in the synovial membrane, further release proinflammatory cytokines such as IL-1β and TNFα [97,98]. IL-1β and TNF-α are the main cartilage degrading cytokines (Figure 2) [98,103]. Both factors are thought to contribute to chondrocyte cell death and stimulate the further release of cartilage-degrading enzymes such as matrix metalloproteinases 1, 2, 9, and 13 (MMP1, 2, 9, 13) and metalloproteinases with thrombospondin motif 4 and 5 (ADAMTS4, 5), which digest proteoglycans and collagen type II that are both important constituents of the cartilage [104,105,106]. The resulting release of collagen and proteoglycan particles in joint space further stimulates the production of additional proinflammatory cytokines, such as IL-18, that propagate further priming and inflammation [76,107]. These processes lead to cartilage fissures with the formation of the new enchondral bone, together with angiogenesis and sensory innervation, which may be closely associated with pain experienced in patients with OA [108]. Once the articular cartilage is damaged, damaged chondrocytes release their content and propagate the inflammatory process to the surrounding synovial membrane, which is considered to play a key role in the pathogenesis of OA [6,109]. Considered a chronic inflammation characteristic of OA, pyroptosis may contribute to pathological changes in all joint-associated structures and account for the loss of cartilage, formation of osteophytes, and synovial membrane inflammation [6].

Another link between inflammasomes and osteoarthritis is the connection of OA to metabolic disorders such as obesity and metabolic syndrome. In obesity, adipose tissue macrophages (ATMs) are differentiated primarily to the M1 subtype and produce proinflammatory cytokines such as IL-1β, TNF-α, IL-6, IL-8, and IL-18 [110,111]. These factors are produced due to inflammasome priming or activation, which may be considered an important factor in OA progression [112]. It has been shown that obese people have greater activity of MMP compared to those with normal weight. The products of MMP activity act as DAMPs, which propagate inflammasome activation that leads to chondrocyte pyroptosis and, thus, cartilage degeneration as the central characteristic of osteoarthritis [113]. Several systemic reviews and meta-analyses have confirmed the association of metabolic syndrome with osteoarthritis development; thus, the term metabolic-syndrome-associated osteoarthritis was coined [114,115]. Since inflammasomes are involved in the low-grade inflammation present in patients with metabolic syndrome (reviewed in [116]), the inflammasome might be considered the key structure in osteoarthritis development in those cases. Furthermore, obesity is often accompanied by metabolic syndrome and an increased amount of uric acid in the body [11,12]. There is increasing evidence that metabolic disorders such as obesity and gout are closely related to a chronic low-grade inflammatory state of the body due to the accumulation of DAMPs that are strong activators of inflammasome [112,117,118].

According to recent studies, inflammasome activation with subsequent pyroptosis of the affected cells may contribute in an important manner to pathological changes in the joint [6,119]. It has been shown that the exposure of chondrocytes to proinflammatory molecules leads to NLRP3 inflammasome activation and subsequent pyroptosis of chondrocytes [120]. Pyroptosis may also be an important mechanism for OA-related pain as proinflammatory cytokines released from pyroptotic cells, particularly IL-1β, as well as depletion of the local population of macrophages, increase the excitability of nociceptors by altering the function of several neuronal ion channels and receptors in the nerve endings around the joint [121,122].

Identification of known inflammasome triggers in OA joints, biomarker evidence, and also connections to known inflammasome-driven diseases suggest the role of inflammasomes and pyroptosis in osteoarthritis development and progression. Nevertheless, additional studies are needed to clarify particular cell type inflammasome activation and pyroptosis in the progression of AO.

## 5. Targeting Different Stages of the Inflammasome Pathway for Treatment of Arthritis

**Figure 3:**
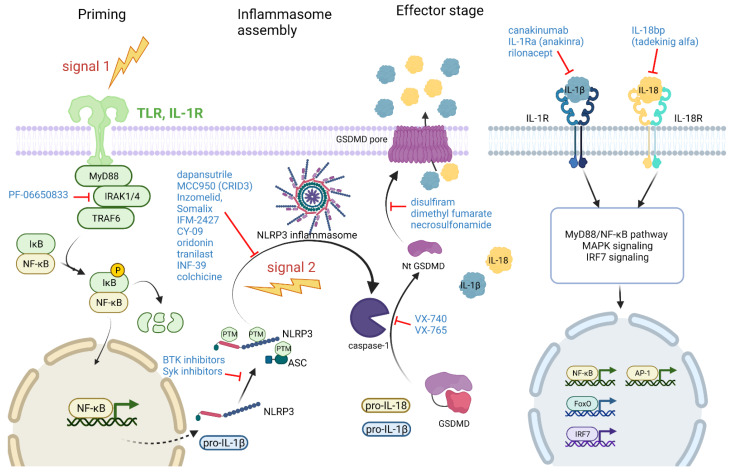
Inflammasome inhibitors may act at different stages of inflammasome activation. Priming, induced by signal 1, through the activation of Toll-like receptors or even IL-1R, leads to the translocation of transcription factor NF-κB and the expression of genes such as pro-IL-1β and NLRP3. During the priming stage and activation stage, inflammasome components are modified by diverse posttranslational modifications (PTMs). Inflammasome activators (signal 2) lead to inflammasome assembly when sensor proteins, e.g., NLRP3, recruit adaptor ASC and pro-caspase-1 molecules. Inflammasome complex formation enables caspase-1 self-activation. Active caspase-1 then cleaves its substrates IL-1β, IL-18, and gasdermin D. Specific molecules were shown to inhibit gasdermin D pore formation. Biological drugs that suppress IL-1R signaling by blocking IL-1β binding are already on the market. Note that compounds that affect priming will also suppress IL-1R and IL-18R signaling.

The inflammasome activation cascade includes the priming stage, inflammasome assembly, the activation stage, and the effector stage (Figure 3). In principle, inhibitory compounds can block any of the stages: priming inhibitors, direct sensor inhibitors, inhibitors of adaptors and caspase-1, as well as inhibitors of effectors: gasdermin D, IL-1β, and IL-18 can be utilized for preclinical and clinical inflammasome suppression. As the NLRP3 inflammasome was shown to be involved in versatile diseases, including RA, the most progress has been made by targeting this pathway, although downstream processes also affect other inflammasomes. Here, we will briefly review inflammasome inhibitors in the context of arthritis, and the reader is also encouraged to read some recent informative reviews [9,123]. For RA treatment, several inflammasome-inhibiting substances have been under clinical investigation. As the only current inflammasome-targeting strategy that is approved worldwide affects the downstream stages (IL-1 signaling), we will start with the effector stages. IL-1β is one of the most important catabolic cytokines linked to arthritis; antagonizing IL-1β signaling has been evaluated for RA therapy in clinical trials. IL-1β signaling can be inhibited either by antibodies against IL-1β (canakinumab, gevokizumab) or by acting on IL-1R signaling with either IL-1 receptor antagonists (IL-1Ra, anakinra), soluble decoy IL-1 receptors (rilonacept), or antibodies targeting IL-1R [124]. The agents that antagonize the action of IL-1R also inhibit IL-1α signaling. Canakinumab, rilonacept, and anakinra are approved by the FDA for the treatment of CAPS. Canakinumab and anakinra are also approved for the treatment of CAPS by EMA, while rilonacept’s marketing license has been withdrawn due to commercial reasons. Canakinumab (Ilaris) is a human anti- IL-1β monoclonal antibody. It completed a large phase III clinical trial evaluating the treatment of cardiovascular disease, named CANTOS, and its clinical development was comprehensively reviewed [125]. The results of the CANTOS trial were published in 2017; in total, 10,061 patients were included [126]. This trial demonstrated that targeting inflammation via inhibiting IL-1β reduced recurrent cardiovascular events without affecting lipid levels. However, the canakinumab-treated group had an increased risk of fatal infection and sepsis [126]. Anakinra is a recombinant form of the endogenous IL-1R antagonist protein that blocks the signaling of IL-1α and IL-1β. In 2001, it was FDA-approved for the clinical indication of moderate-to-severe RA, with prior failure of at least one other disease-modifying antirheumatic drug therapy [127]. Nevertheless, it is an uncommonly prescribed drug due to its moderate efficiency when compared to more efficient anti-TNFα therapies of RA [128]. Inflammasome activation results in IL-18 activation, which also contributes to the pathogenesis of arthritis. A recombinant IL-18 binding protein (tadekinig alfa) showed promising results in two patients with Still’s disease [129].

Another effector is gasdermin D. Blocking gasdermin D pore formation not only inhibits pyroptosis but also the release of IL-1β from living cells [25,26]. Preclinical studies suggest that metabolites and ROS influence gasdermin D’s pore-forming activity [130,131]. Targeting C192 might be a viable strategy for the manipulation of pore formation and can be achieved by two repurposed drugs, disulfiram and dimethyl fumarate, and necroptosis inhibitor necrosulfonamide [130,132,133].

Inhibiting caspase-1 activity is another strategy that is common to different inflammasomes; however, despite extensive research, none of the candidates so far have provided a good efficacy and safety profile in clinical studies [123,134]. Among caspase-1 inhibitors, VX-740 and VX-765, after being efficient in animal models, entered clinical studies. VX-740 was tested in a phase IIb trial for RA; however, the trial was stopped due to liver toxicity observed in animals (not humans) after prolonged exposure. VX-765 completed clinical phase II trials for epilepsy [135] and psoriasis [136], and the clinical trial phase IIB for epilepsy was discontinued despite the drug appearing safe [137]. Pyroptosis-released ASC specks can propagate inflammation [28,29]. Nanobodies targeting ASC disassemble ASC specks and ameliorate inflammation in mouse models of gout and antigen-induced arthritis [30].

In the past two decades, several small-molecule inhibitors that target the NLRP3 sensor directly have been developed. The main lead compound, MCC950 (CRID3, CP456,773), was first tested in a clinical trial for RA; however, some liver toxicity was observed [138]. In 2015, Coll and colleagues demonstrated that MCC950 potently and selectively inhibits the NLRP3 inflammasome [139]. MCC950 binds directly to the NACHT domain of NLRP3 [140,141,142]. Recently, Hochheiser et al. solved the structure of inactive NLRP3 decamers in a complex with MCC950 [43], showing that MCC950 interacts with different segments of the NACHT domain, stabilizing the NLRP3 in an inactive conformation [43]. Several similar compounds have entered clinical trials. Phase I clinical trials with MCC950 derivative CNS-penetrant Inzomelid [143] and peripherally restricted Somalix [143] (Inflazome, now Roche) were concluded, demonstrating the safety and tolerability of the drugs.

Another peripherally distributed drug, IFM-2427 (DFV890) (IFM Tre/Novartis), has been registered for a phase II clinical study in patients with knee osteoarthritis [144].

Preclinical studies have demonstrated the potential of several other direct NLRP3 NACHT-binding compounds, CY-09 [145], oridonin [146], and tranilast [147], or NLRP3 ATPase inhibitors such as INF-39 [148]. Tranilast has been approved for the treatment of allergic disease in Japan and South Korea and has entered a phase II clinical trial for CAPS [143].

Dapansutrile or OLT1177 (Olatec) is a β-sulfonyl nitrile that has been shown to selectively inhibit NLRP3 inflammasome activation in a preclinical study [149]. OLT1177 reduced NLRP3 ATPase activity and prevented NLRP3-ASC interaction. It advanced into clinical studies. In a phase II clinical study for patients with acute gout, a short treatment with dapansutrile decreased the pain comparably to NSAIDs and prednisolone. At the doses used, no toxicity was observed [150].

In addition to the above-described direct NLRP3 inhibitors, it is also possible to suppress inflammasome activation by acting on posttranslational modifiers of inflammasome components or by affecting priming. Particularly, the expression of pro-IL-1β is enhanced in the priming stage. Diacerein has been approved for osteoarthritis treatment due to its inhibitory action on IL-1β expression and release [151]. Due to side effects such as diarrhea and hepatobiliary disorders, it is contraindicated in older patients and patients with liver disease [143,151]; due to the lack of data and poor quality of the studies, it is not recommended for OA treatment by the expert societies EULAR and ACR and only conditionally recommended by ESCEO [151]. Considering the indirect inhibitors of NLRP3, Bruton’s tyrosine kinase (BTK) inhibitors, such as ibrutinib and evobrutinib, are being explored for the treatment of autoimmune diseases [152]. BTK was shown to be directly involved in NLRP3 inflammasome activation [153,154,155]. Fenebrutinib has successfully passed a phase II clinical trial as a selective, reversible BTK inhibitor for the treatment of patients with RA who have a poor response to methotrexate therapy [156]. IRAK4 signaling is involved in the priming stage as well as in the effector stage by supporting IL-1R signaling. Through the Myd88/IRAK pathway, NF-κB mediates the transcription upregulation of many inflammasome components, including IL-1β [32]. A Phase IIb clinical study with the IRAK4 inhibitory substance called PF-06650833 (Pfizer) in patients with RA was recently completed. It demonstrated an apparent efficacy, changing the disease activity measured with a simplified disease activity index [157]. Another potentially interesting signaling pathway involves spleen tyrosine kinase (Syk), which, through the signaling cascade, also upregulates the production of inflammasome components, including IL-1β. One of the substances inhibiting Syk is fostamatinib (Tavalisse), which has passed a phase II clinical trial and proceeded to the phase III clinical trial OSKIRA (Oral Syk Inhibition in Rheumatoid Arthritis). In OSKIRA, fostamatinib showed statistically significant improvement in RA activity and fulfilled safety requirements, but it has not undergone regulatory procedures before use [158] (reviewed in [123]).

Colchicine has been used since antiquity for the treatment of swollen joints. Colchicine, by acting on microtubules, inhibits the canonical NLRP3 inflammasome and pyrin inflammasome activation [159,160]. It is still used for the treatment of gout and pyrin inflammasome-mediated familial Mediterranean fever [161].

Current studies demonstrate the major involvement of the NLRP3 inflammasome in secondary arthritic diseases. Multiple levels of evidence also point to the NLRP3 inflammasome as an important culprit in the development of primary osteoarthritis. As some data suggest that other (non-NLRP3) inflammasomes are involved, we expect that future studies will better define the source of IL-1β, one of the most important cytokines in OA progression. The development of inflammasome inhibitors that act at different stages of inflammasome activation, as well as present different levels of specificity, is a very vibrant topic of preclinical and clinical research, fueling optimism that some of those drugs, particularly those that are already marketed for other conditions, could also be used for the treatment of osteoarthritis in the near future.

## Data Availability

Not applicable.
